# COGNITIVE BEHAVIORAL TREATMENT OF INSOMNIA SLOWS THE PACE OF BIOLOGICAL AGING: RESULTS FROM AN RCT IN OLDER ADULTS

**DOI:** 10.1093/geroni/igad104.0678

**Published:** 2023-12-21

**Authors:** Judith Carroll

**Affiliations:** University of California, Los Angeles, Los Angeles, California, United States

## Abstract

Insomnia in late life has been linked to accelerated biological aging, presenting a behaviorally modifiable target to slow the pace of aging. We previously demonstrated that treatment of insomnia in older adults using cognitive behavioral therapy for insomnia (CBT-I) reduced the expression of the cellular senescence marker, p16INK4a, in peripheral blood mononuclear cells (PBMC). Next, we tested whether this treatment for insomnia would alter the biological pace of aging, estimated from the DNA methylation derived DunedinPACE, as compared to a sleep education therapy (SET), an active comparator condition. Participants 60+ years old with insomnia were enrolled in a randomized controlled trial and assigned to CBT-I (n=49) or SET (n=45). Epigenetic data were obtained from DNA extracted from blood collected prior to initiation of treatment (baseline) and again 18-24 months later. A mixed linear models adjusting for age, sex, BMI, and education revealed a significant treatment by time interaction (p=0.003). Receipt of CBT-I was associated with a decline in the epigenetic pace of aging, while those receiving SET had an increase in their pace of aging. Findings indicate that a behavioral intervention to improve insomnia was effective at slowing the pace of biological aging among older adults. Future research will be needed to replicate these findings in other age groups and specific populations with heightened risk for accelerated aging.

